# Spatial and temporal localization of homogalacturonans in *Hyacinthus orientalis* L. ovule cells before and after fertilization

**DOI:** 10.1007/s00299-014-1690-8

**Published:** 2014-10-08

**Authors:** Katarzyna Niedojadło, Malwina Hyjek, Elżbieta Bednarska-Kozakiewicz

**Affiliations:** Department of Cell Biology, Faculty of Biology and Environment Protection, Nicolaus Copernicus University, Lwowska 1, 87-100 Toruń, Poland

**Keywords:** Homogalacturonan (HG), Pectin, Ovule, Embryo sac, *Hyacinthus orientalis*

## Abstract

*****Key message***:**

**The composition of homogalacturonans (HGs) in the ovule and the female gametophyte cell walls was shown to be rearranged dynamically during sexual reproduction of** ***H. orientalis***.

**Abstract:**

In angiosperms, homogalacturonans (HGs) play an important role in the interaction between the male gametophyte and the pistil transmitting tract, but little is known about the participation of these molecules at the final stage of the progamic phase and fertilization. The aim of our study was to perform immunocytochemical localization of highly (JIM7 MAb) and weakly (JIM5 MAb) methyl esterified and Ca^2+^-associated HG (2F4 MAb) in the ovule and female gametophyte cells of *Hyacinthus orientalis* before and after fertilization. It was found that pollination induced the rearrangement of HG in (1) the micropylar canal of the ovule, (2) the filiform apparatus of the synergids, and (3) the region of fusion between sperm cells and their target cells. Fertilization led to further changes in pectin composition of these three regions of the ovule. A new cell wall was synthesized around the zygote with a characteristic pattern of localization of all examined HG fractions, which we called “sporoderm-like”. The developing endosperm prepared for cellularization by synthesizing highly methyl-esterified HG, which was stored in the cytoplasm. Pollination- and fertilization-induced changes in the composition of the HG in the micropyle of the ovule and the apoplast of female gametophyte cells are discussed in the context of: (1) micropylar pollen tube guidance, (2) preparation of the egg cell and the central cells for fusion with sperm cells, and (3) the polyspermy block.

## Introduction

The plant cell wall is a highly complex and dynamic structure composed of polysaccharides, structural proteins and phenolic compounds (Somerville et al. 2004; Cosgrove 2005; Wolf and Greiner 2012). Polysaccharides are often grouped into three functional categories: celluloses, hemicelluloses, which mainly consist of xyloglucan (XG) and small amounts of glucuronoarabinoxylan, and pectins, which are a family of galacturonic acid-rich polysaccharides including homogalacturonan (HG), rhamnogalacturonan I (RG-I), rhamnogalacturonan II (RG-II) and xylogalacturonan (XGA). The most common structural model of the cell wall depicts a cellulose–hemicellulose network embedded in a pectin matrix (Mohnen 2008; Dick-Pérez et al. 2011). The interactions between these polysaccharides control important aspects of plant development including cell adhesion, wall extensibility, wall porosity and the mediation of defense responses (Krupkova et al. 2007; Mohnen 2008; Caffall and Mohnen 2009; Wallace and Anderson 2012). HGs are polymerized and methyl esterified in the Golgi apparatus and secreted to the cell wall in a highly methyl-esterified state (Zhang and Staehelin 1992; Sterling et al. 2006), where they can undergo de-esterification by cell wall-associated pectin methyl esterases (PMEs). The removal of methyl groups dramatically alters physical properties of the polymers. During this process, free carboxylic acid groups are created, and methanol and protons are released (Wolf et al. 2009; Wolf and Greiner 2012). After de-methyl esterification, HG can form Ca^2+^-pectate cross-linked complexes, the so called “egg-boxes” (Grant et al. 1973), which indicate a denser and more inextensible cell wall (Peaucelle et al. 2012). The controlled lysis of this category of pectin may regulate the release of free Ca^2+^ ions, which participate in various physiological processes (Wolf et al. 2009). Conversely, as a result of PME activity, the protons decrease local cell wall pH and promote the activity of cell wall hydrolases such as polygalactouronases and pectin/pectate lyases, which lead to cell wall loosening. The de-esterification of HGs can lead to both cell wall stiffening through the creation of egg boxes as well as to enzymatic degradation of pectin. Hence, the methyl esterification status of HGs can have dramatic consequences on cell wall texture and mechanical properties, thereby regulating cellular growth and shape. The control of methyl esterification status of HGs also induces formation of signaling molecules with consequential effects on development. Pectic oligogalacturonides (OGAs), small breakdown products of HGs, have been shown to act as signaling molecules, both as elicitors during pathogen attack and as hormone-like compounds that counteract the effects of auxin during plant development (Ridley et al. 2001; Wolf et al. 2009).

The functions of HGs in sexual reproduction are now well established. HGs play an important role in pollen–pistil interactions before double fertilization. Pollination induces several changes in both distribution and metabolism of highly and weakly methyl-esterified HGs localized in the stigma and the style transmitting tract. This process has been indicated as a mechanism of forming an optimal Ca^2+^ environment at the site of pollen grain germination and pollen tube growth in vivo (Lenartowska et al. 1997, 2001; Bednarska et al. 2005; Dresselhaus and Márton 2009; Lenartowska et al. 2011; Dresselhaus and Franklin-Tong 2013). The role of the extracellular matrix (ECM) in this process was studied in various organisms including *Lilium longiflorum* (Zhao et al. 2004), *Nicotiana* (Ge et al. 2009), *Brassica napus*, *Helianthus annuus*, *Gossypium hirsutum* (Zhang et al. 1995, 1997; Ge et al. 2007), *Petunia hybrida* (Lenartowska et al. 2001), *Trithuria* (Costa et al. 2013), and *Olea europaea* L. (Suárez et al. 2013). Additionally, pectin methyl esterases and polygalacturonases secreted by growing pollen tubes lead to HGs degradation, and this process likely results in ECM loosening and facilitation of tubes penetration through the pistil (Lenartowska et al. 2001; Wolf et al. 2009; Hepler et al. 2013). Ca^2+^ in the stigma is taken up by germinating pollen grains (Bednarska 1991) and accumulates in the apical zone of the pollen tube, forming a characteristic tip-to-base gradient (Rathore et al. 1991; Miller et al. 1992; Pierson et al. 1996; Dresselhaus and Márton 2009). Calcium ions are also involved in adhesion through the formation of “egg-box” complexes between weakly methyl-esterified HGs of the sporoderm and the stigmatic surface (Bednarska et al. 2005) as well as the stylar transmitting tract (Lenartowska et al. 2001). Unesterified HG has been demonstrated to interact with a cysteine-rich adhesion (SCA) protein in *L. longiflorum* to participate in adhesion between stylar transmitting cells and pollen tubes (Mollet et al. 2000). Moreover, studies on the stigmatic cuticle of *Amborella*, *Illicium*, *Trimenia*, *Acorus* and two species within *Chloranthaceae* revealed that the hydrophilic molecular network formed by HGs and AGPs enhances permeability and acts as a pathway for the movement of water and other molecules that function in cellular interactions between the stigma and pollen (Hristova et al. 2005; Sage et al. 2009). Methyl-esterified HG is also presumed to play a role in hydration and stabilization of transmitting tissue’s ECM (Carpita and Gibeaut 1993; Sage et al. 2009).

Until now, there has been no knowledge of the role of HGs at the final stage of progamic phase. Inside the ovary, a pollen tube entering from the funiculus must first find its way to the micropyle to reach the embryo sac and then target one of two synergid cells before bursting to release two sperm cells (Higashiyama et al. 2001; Yagedari and Drews 2004; Higashiyama and Hamamura 2008; Kessler and Grossniklaus 2011). For many years it has been postulated that Ca^2+^ ions could be responsible for this chemoattraction (Mahló and Trewavas 1996; Hepler 1997; Zhang and Cass 1997; Hepler et al. 2012) or are only a part of an attractant cocktail of molecules (Dresselhaus and Márton 2009; Dresselhaus and Franklin-Tong 2013) for growing pollen tubes. Recent studies on *Torenia fournieri* indicate that synergids can guide elongating pollen tubes towards the embryo sac via extracellular secretion of polypeptide, species-specific small chemoattractant molecules (Okuda et al. 2009). Thus, calcium ions are now considered a relevant nutrient factor for proper pollen tube elongation rather than specific guiding signal. It has been shown, that pollen germination and pollen tube growth is subject to Ca^2+^ storage sites in the pistil in many species (Ge et al. 2007). The precise mechanism that regulates calcium level and HGs distribution in the embryo sac remains unrevealed. Elevated levels of HGs were identified in the fibrillar filiform apparatus of the synergids (Huang and Russel 1992). Additionally, calcium distribution studies in ovules indicated that the micropyle and synergid filiform apparatus accumulated abundant amounts of free Ca^2+^ (Chaubal and Reger 1992a; Tian and Russel 1997; Higashiyama et al. 2003; Dumas and Gaude 2006).

Our studies were aimed at the immunocytochemical localization of HGs in *H. orientalis* ovules and embryo sacs before and after fertilization. We also analyzed ovules during the progamic phase when the pollen tubes had reached approximately three-quarters of the style length and had not entered the female gametophyte yet. This study is the first report showing changes in the distribution of HG epitopes at this stage of *Angiospermae* female gametophyte development. We used three anti-pectin monoclonal antibodies, which have been extensively used in immunofluorescence and immunogold localization studies: JIM5 against weakly methyl-esterified homogalacturonic acid epitopes, JIM7 against the epitopes of highly methyl-esterified (mainly by methyl groups) forms of the acid and MAbs 2F4, which binds specifically to calcium-crosslinked dimeric pectic chains. It binds to HGs with degrees of methyl esterification of up to 40 % (Liners et al. 1989; Knox et al. 1990; Knox 1997; Willats et al. 2000; Lenartowska et al. 2001; Abreu and Oliveira 2004; Rafińska and Bednarska 2011). The results are discussed in relation to the possible roles of HGs in creating an optimal environment for pollen tubes growing in vivo during successive stages of *H. orientalis* female gametophyte development and fertilization.

## Materials and methods

### Plant material

Commercial cultivars of *H. orientalis* L. variety Pink Pearl were used in the investigation. The ovules were mechanically isolated from flowers at anthesis and 8, 48, and 96 h after manual cross-pollination. The growth of the pollen tubes was checked in isolated pistils, which were cut, placed in 0.01 % aniline blue and examined using fluorescence microscopy.

### Sample processing

Immediately after isolation, ovules were fixed in 4 % paraformaldehyde (Polyscience) and 0.25 % glutaraldehyde (Sigma Aldrich), dehydrated in increasing ethanol concentrations containing 10 mM dithiothreitol (DTT) (Fermentas) and embedded in BMM resin at −20 °C under UV light for polymerization (in butyl methacrylate, methyl methacrylate, 0.5 % benzoyl ethyl ether (Fluka) and 10 mM DTT). The embedded material was cut on a Leica UCT ultramicrotome into longitudinal, serial semi-thin sections (1, 5 µm) that were placed on microscope slides coated with biobond (British Biocell). Before immunocytochemical reactions, the resin was removed with two changes of acetone and washed twice in water and PBS at pH 7.2.

To perform immunolocalization of highly and weakly esterified HG, the sections were treated with blocking solution (PBS at pH 7.2 and 2 % BSA) for 1 h at room temperature and then incubated with rat JIM5 and JIM7 primary antibodies (Plant Probes) at 1:50 in PBS at pH 7.2 with 0.2 % BSA overnight at 4 °C. JIM7 antibody binds HGs showing 15–80 % esterification, containing epitopes composed of methyl-esterified residues with adjacent or flanking unesterified residues (Willats et al. 2000; Clausen et al. 2003). JIM 5 recognizes HG showing 31–40 % esterification, containing epitopes composed of four or more contiguous unesterified residues adjacent to or flanked by residues with methyl-ester groups (Willats et al. 2000; Clausen et al. 2003). After washing with PBS, the sections were incubated with Cy3-conjugated affinipure rabbit, anti-rat secondary antibodies (Jackson Immunoresearch) diluted 1:100 in PBS at pH 7.2 with 0.2 % BSA for 1 h at 37 °C. To detect Ca^2+^-bound HGs, sections were treated with 0.1 % BSA in 20 mM Tris–HCl at pH 8.2 for 10 min at room temperature and incubated with mouse 2F4 primary antibodies (Plant Probes) at 1:50 in 0.1 % BSA in 20 mM Tris–HCl at pH 8.2 overnight at 4 °C. After washing with 20 mM Tris–HCl at pH 8.2, sections were incubated with Cy3-conjugated affinipure rabbit, anti-mouse secondary antibodies (Sigma) diluted 1:100 in 20 mM Tris–HCl at pH 8.2 for 1 h at 37 °C. Then, sections were washed in PBS at pH 7.2 or 20 mM Tris–HCl at pH 8.2. DNA was stained with 4 pg/ml DAPI (4′,6-diamidino-2-phenylindole, Fluka) solution in water for 5 min. Next, sections were washed in distilled water, dried at room temperature and covered with 0.5 % w/v *N*-phenylenediamine.

To perform control reactions, primary antibodies were omitted. The controls showed no labeling; only strong autofluorescence of the chalazal region of the embryo sac wall was visible.

### Quantitative analysis

Image analysis was performed on serial semi-thin sections after immunofluorescence staining (methyl-esterified, de-methyl-esterified, and calcium-bound HGs), with each reaction step performed using consistent values of temperature, incubation times, and concentration of primary and secondary antibodies. Quantitative analysis of fluorescence intensity for immunofluorescence staining was carried out for 5–7 each cell types (5 sections per cell) from each development stage. All measurements were conducted at the same magnification, field area (controlled with a shutter), and positioning of the fiber optics cable. Camera settings were kept constant for exposure time, gain and offset. Lucia G software was used to determine the average µm^3^ signal intensity of each studied cell compartment and is expressed in a.u. (arbitrary unit of fluorescence intensity). For all antigens and developmental stages, the obtained data were corrected for background autofluorescence as determined from negative control signal intensities.

To test differences among multiple samples (groups, i.e., antigen level in different stages), a Kruskal–Wallis ANOVA test was used. Statistical data and graphs were created using Microsoft Excel 2007 software.

## Results

The immunocytochemical localization of HGs revealed different distribution of the epitopes recognized by JIM7 (highly esterified HG), JIM5 (weakly esterified HG) and 2F4 (dimerized, Ca^2+^-linked HG) antibodies in somatic cells of *H. orientalis* ovules and in embryo sac cells before and after fertilization.

### Immunolocalization of HGs in the ovule

**Fig. 1:**
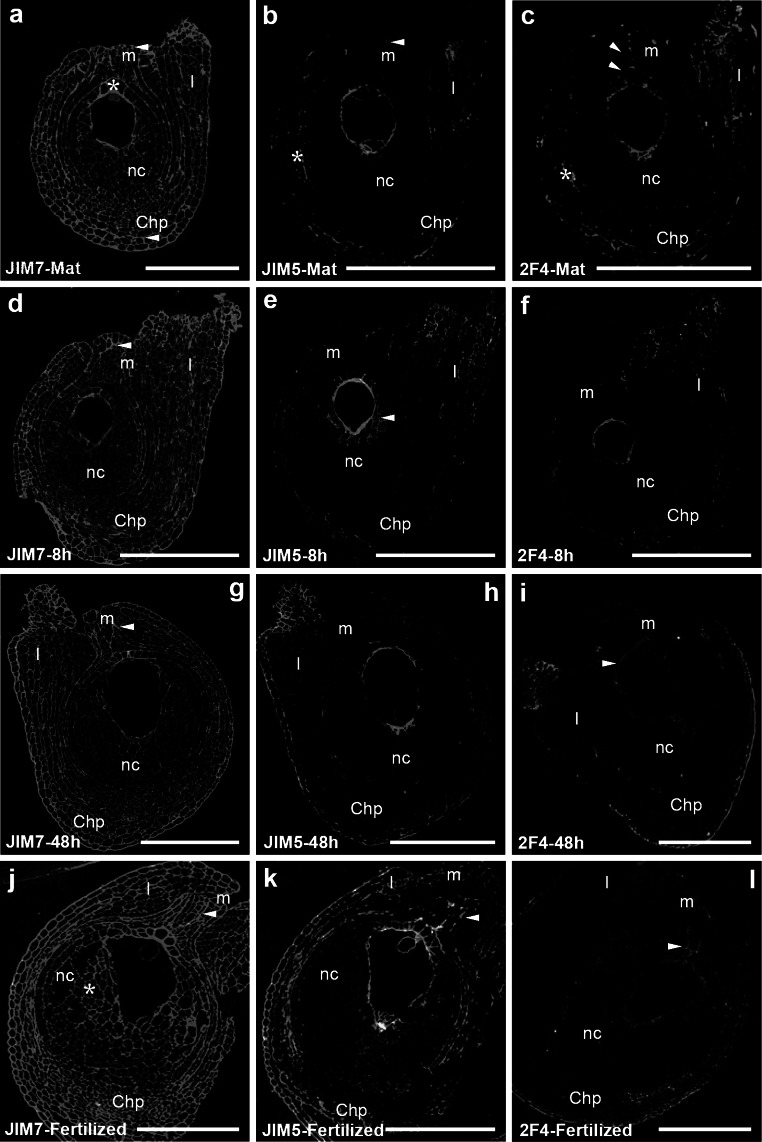
Immunofluorescence localization of HGs in *H. orientalis* ovule with JIM7, JIM5 and 2F4 MAbs. Longitudinal sections of the mature ovule (**a**–**c**), 8 h (**d**–**f**) and 48 h (**g**–**i**) after pollination (progamic phase), and after fertilization (**j**–**l**) are shown. JIM7 epitopes were localized in almost all walls of somatic cells and in the wall of gametophyte during all stages of development. The highest fluorescence signal was visible at the micropylar pole and in the micropylar canal (**d**, **g**, **j**, *arrowheads*). Lower level of JIM5 labeling in the micropylar region of mature ovule was noted (**b**, *arrowhead*). Differences in distribution of weakly esterified HG were observed after pollination. Level of JIM5 labeling in the region surrounding the embryo sac increased (**e**, *arrowhead*), and after fertilization, strong fluorescence signal was present also at the micropylar pole of the ovule (**k**, *arrowhead*). In mature ovule, heterogeneous fluorescence of 2F4 in the micropylar canal (**c**, *arrowheads*) and around the cells of micropylar region of nucellus was high (**c**). During the progamic phase, 2F4 epitopes were still localized in this region of the ovule (**f**, **i**), but the level of fluorescence was much lower than before pollination (**c**). After fertilization, distinct changes of epitopes recognized by 2F4 were visible. Strong fluorescence in cells surrounding the micropylar canal and the micropyle was observed (**l**, *arrowhead*). Labeling of the embryo sac cells is not visible due to low magnification of the images. *m* micropyle, *nc* nucellus, *Chp* chalazal pole, *I* integument, *Bars* 100 µm

In somatic cells of mature ovules, signal from highly methyl-esterified HG was predominant. Strong and homogeneous signal of fluorescence detected by JIM7 was observed in cell walls of the integuments. The highest signal was observed in the micropylar and chalazal regions of the ovule (Fig. 1a, *arrowheads*). In nucellus, fluorescence detected by JIM7 was lower, and higher accumulation of methyl-esterified HG was identified only in the single layer of cells surrounding the gametophyte at the micropylar pole (Fig. 1a, *asterisk*). Levels of weakly esterified HGs (Fig. 1b) and Ca^2+^-bound HGs (Fig. 1c) in somatic cells of the ovule were distinctly lower. Weak fluorescence indicating localization of JIM5 and 2F4 antibodies was observed in cell walls of integuments (Fig. 1b, c, *asterisk*). In the ovule’s micropylar canal, only epitopes recognized by 2F4 were visible as numerous small clusters (Fig. 1c, *arrowheads*). The nucellus was almost completely devoid of signal. In the wall enclosing the gametophyte, all the studied fractions of HGs were observed (Fig. 1a–c).

During the progamic phase, i.e. 8 and 48 h after pollination, no significant changes had occurred in JIM7 labeling of the *H. orientalis* ovule. A strong fluorescence signal was still localized in cell walls of integuments and in the embryo sac wall (Fig. 1d, g). In contrast, variation in the localization of weakly esterified HG was observed during the progamic phase. Eight hours after pollination, the epitopes recognized by JIM5 were localized in the walls of somatic cells adjacent to the female gametophyte (Fig. 1e, *arrowhead*). In the ovule’s micropylar canal, single small foci of fluorescence from JIM5 were sometimes visible. A similar pattern of weakly esterified HG distribution was observed 48 h after pollination (Fig. 1h). Additionally, during the progamic phase, relevant differences in distribution of Ca^2+^-associated HGs were also observed. Levels of 2F4 labeling in the micropylar canal (Fig. 1f, i) and the gametophyte wall (Fig. 1i,* arrowhead*) were much lower than before pollination.

After fertilization, the pattern of localization of highly esterified HG in the ovule underwent significant changes. Strong signal detected with JIM7 was still observed in cell walls of integuments (Fig. 1j). In the walls of cells at the micropylar pole of the ovule and in the somatic cells enclosing the embryo sac, an increase in the intensity of fluorescence was observed (Fig. 1j, *arrowhead*). High accumulation of HGs recognized by JIM7 also occurred in the ECM of nucellus (Fig. 1j, *asterisk*). The level of weakly esterified HGs was also higher than before fertilization (Fig. 1k). Strong signal from JIM5 antibody was still present in the embryo sac wall; however, after fertilization, JIM5-reactive epitopes were also localized in the walls of somatic cells enclosing embryo sac’s micropylar pole, as well as in the micropylar canal (Fig. 1k, *arrowhead*). Distinct changes were visible for the epitopes recognized by 2F4 (Fig. 1l). Compared to before fertilization, slightly stronger fluorescence in walls of cells surrounding the micropylar canal and at the micropyle was observed (Fig. 1l, *arrowhead*). 2F4 labeling was also identified in embryo sac wall (Fig. 1l).

### Immunolocalization of HGs in embryo sac cells

**Fig. 2:**
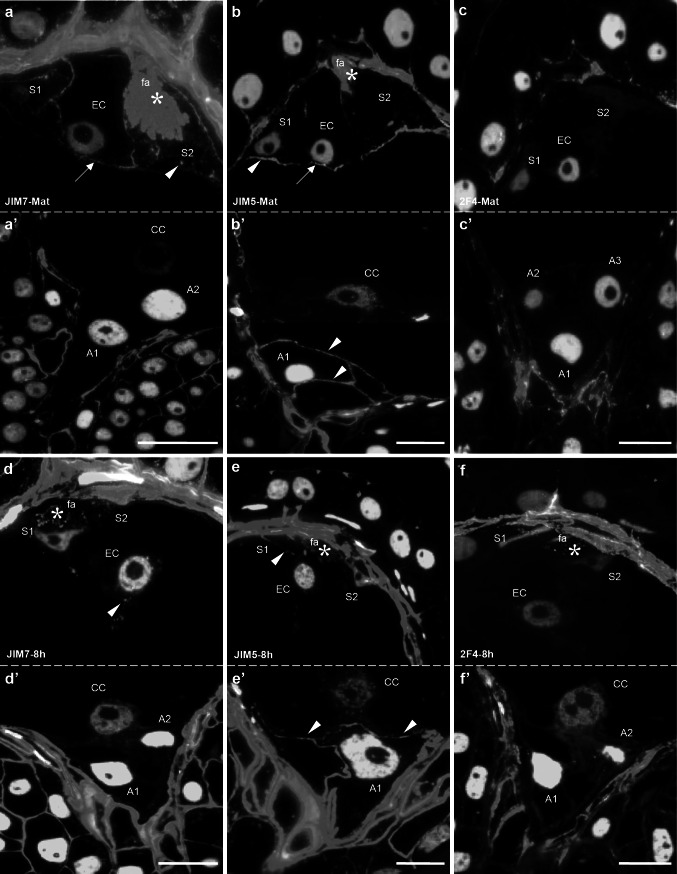
Immunofluorescence localization of epitopes recognized by JIM7, JIM5 and 2F4 in the *H. orientalis* mature embryo sac (**a**, **a**′**, b**, **b**′, **c**, **c**′) 8 h after pollination—progamic phase (**d**, **d**′, **e**, **e**′, **f**, **f**′). In the mature embryo sac, HGs with high degree of esterification (JIM7) and with low degree of esterification (JIM5) were observed in the filiform apparatus (**a**, **b**, *asterisks*) and the chalazal ends of the synergid walls (**a**, **b**, *arrowheads*). In contrast, the filiform apparatus was completely devoid of 2F4 labeling (**c**). Only single small clusters of fluorescence were visible in synergid walls. In mature egg cells, labeling from both JIM7 and JIM5 was visible (**a**, **b**, *arrows*). In antipodal cells walls, only weakly esterified HG (JIM5) was localized (**b**′, *arrowheads*). During the progamic phase, the level of labeling by JIM7 in the filiform apparatus and synergid walls was lower than before pollination (**d**, *asterisk*). In contrast, JIM5 labeling of filiform apparatus extended to the whole structure, and the signal was present in both filiform apparatus (**e**, *asterisk*) and synergid walls (**e**, *arrowhead*). Eight hours after pollination, the egg cell wall was completely devoid of fluorescence from both JIM5 (**e**) and 2F4 (**f**). In the antipodal cells, before and after pollination, only epitopes recognized by JIM5 were observed (**b**′, **e**′, *arrowheads*). No fluorescence signal after JIM7 (**a**′, **d**′) and 2F4 (**c**′, **f**′) labeling was visible. *S1*, *S2* synergid cells, *EC* egg cell, *CC* central cell, *A1*, *A2*, *A3* antipodal cells, *fa* filiform apparatus, *Bars* 20 µm

In mature embryo sac cells of *H. orientalis*, the highest signal of methyl-esterified HGs was observed in synergid’s filiform apparatus (Figs. 2a, *asterisk*, 4c). Lower signal of JIM7 labeling was also visible in synergid walls, which were adjacent to the egg cell and to the embryo sac wall (Fig. 4b). However, in synergid walls adjacent to central cell, only single foci of fluorescence were observed (Fig. 2a, *arrowhead*). In the filiform apparatus, epitopes recognized by JIM5 were present (Fig. 4f), but the signal was not as evenly distributed compared to JIM7 and was restricted to the most micropylar region of this structure (Fig. 2b, *asterisk*). High but discontinuous signal from weakly esterified HGs was also localized in synergids walls, separating them from the egg cell (Fig. 2b). Within egg cell wall, labeling by both JIM7 (Fig. 2a, *arrow*) and JIM5 (Fig. 2b, *arrow*) was observed. In the chalazal ends of egg apparatus, the signal from JIM7 was weak and discontinuous (Fig. 2a, *arrow*). Additionally, this fraction of HG was not observed in the cytoplasm of the egg cell. At this stage of development, HG recognized by 2F4 was not detected within any of the egg apparatus cell (Figs. 2c, 4g–i). In antipodal cell walls, only epitopes recognized by JIM5 were observed (Fig. 2b′, *arrowheads*). The labeling from JIM7 (Fig. 2a′) and 2F4 (Fig. 2c′) was not visible.

During the progamic phase, the immunocytochemical detection of HGs revealed relevant differences in distribution of highly esterified HG. Eight hours after pollination, in synergid cells, the level of labeling by JIM7 was lower in comparison to the period before pollination (Fig. 2d, *asterisk* compared with Fig. 2a; Fig. 4b). De-esterified HG was present primarily in the filiform apparatus (Figs. 2e, *asterisk*, 4f). Also, very low signal from 2F4-reactive epitopes was also observed in this structure (Figs. 2f, *asterisk*, 4i).

**Fig. 3:**
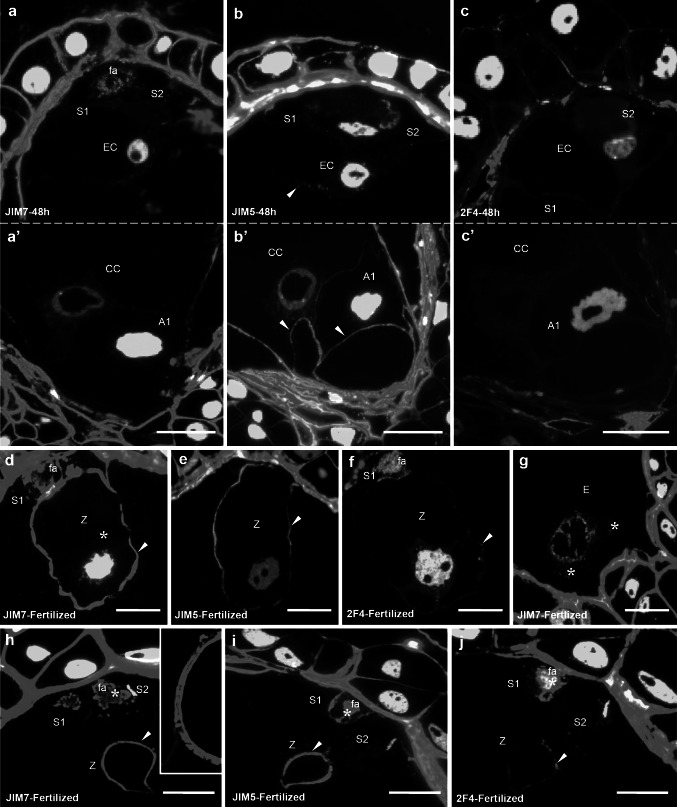
Immunofluorescence localization of epitopes recognized by JIM7, JIM5 and 2F4 in *H. orientalis* embryo sac 48 h after pollination (progamic phase) (**a**, **a**′, **b**, **b**′, **c**, **c**′) and after fertilization (**d**–**j**). Forty-eight hours after pollination, the level of labeling by JIM7 in the filiform apparatus and the walls of the synergid cells was lower than before pollination (**a**). The level of unesterified HG (JIM5) was high and similar to that of 8 h after pollination (**b**). In synergid cells, Ca^2+^-associated HGs were not observed (**c**). Forty-eight hours after pollination, labeling by JIM5 (**b**) and JIM 7 (**a**) was visible in the egg cell. No calcium-bound HGs were detected (**c**). After fertilization, labeling by JIM5, JIM7 and 2F4 antibodies were localized in the filiform apparatus of degenerating synergid cells (**d**–**f**, **h**–**j**, *asterisks*). The zygote was surrounded by thick wall, where comparable levels and specific distribution of labeling by JIM7 (**d**, **h**, *arrowheads*) and JIM 5 (**e**, **i**, *arrowheads*) were observed. The fluorescence signal after 2F4 labeling was lower (**f**, **j**, *arrowheads*). In the zygote cytoplasm and in the cytoplasm surrounding endosperm nuclei, small clusters of fluorescence recognized by JIM7 were present (**d**, **g**, *asterisks*). *S1*, *S2* synergid cells, *EC* egg cell, *fa* filiform apparatus, *Z* zygote, *E* endosperm, *Bars* 20 µm

**Fig. 4:**
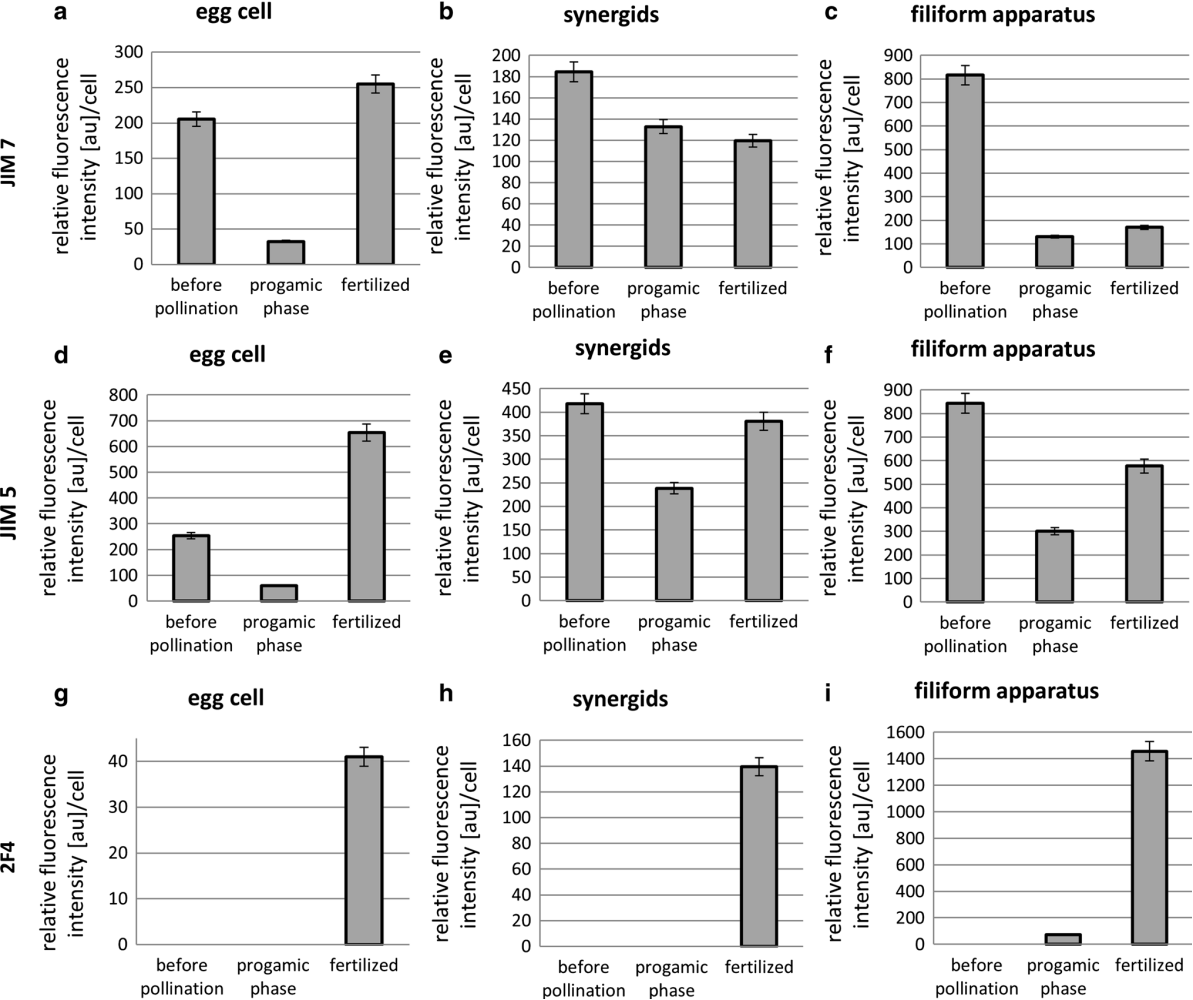
Histograms illustrating changes in relative intensities of JIM7 (**a**–**c**), JIM 5 (**d**–**f**) and 2F4 (**g**–**i**) fluorescence in egg apparatus cells walls before and after fertilization. *Error bars* represent standard deviation of the mean

During the progamic phase, the labeling of esterified HG around the egg cell was generally significantly weaker than that observed before pollination (Fig. 4a). Eight hours after pollination, only a single spot of fluorescence was visible after JIM7 labeling (Fig. 2d, *arrowhead*). Unesterified HG (Fig. 2e) and HG associated with calcium (Fig. 2f) were not detected in this cell (Fig. 4 d, g). Forty-eight hours after pollination, when the pollen tubes grew into the ovary, in the ECM of the egg cell, small clusters of fluorescence recognized by JIM5 (Fig. 3b, *arrowhead*) and JIM7 (Fig. 3a) were observed, but the level of signal was significantly lower than before pollination (Fig. 4a, d). 2F4 labeling was not observed (Fig. 3c). Throughout the progamic phase, only unesterified HG (JIM5) was visible in the antipodal cell walls (Figs. 2e′, 3b′,* arrowheads*).

After fertilization, changes in the pattern of distribution of all analyzed HGs categories within *Hyacinthus* egg apparatus were observed. In the filiform apparatus of degenerating synergid, highly esterified HGs (JIM7) (Fig. 3h, *asterisk*) and weakly esterified HGs (JIM5) (Fig. 3i, *asterisk*) were still present. However, in this structure, significantly higher signal from 2F4-reactive epitopes compared with before fertilization was visible (Figs. 3j, *asterisk*, 4i). Significant changes in HGs localization were also observed in the zygote. In comparison to the egg cell during progamic phase, very high level of fluorescence detected by JIM7 was observed in the zygote wall (Figs. 3d, *arrowhead*, 4a). Additionally, single clusters of signal in the zygote’s cytoplasm were observed (Fig. 3h,* asterisk*). In the cell wall, epitopes recognized by JIM5 were present (Fig. 3e, *arrowhead*), while 2F4 labeling was very weak (Figs. 3f,* arrowhead*, 4g). Shortly after fertilization, the zygote was surrounded by a thick wall, in which relatively high levels of highly esterified (Fig. 3h, *arrowhead*) and unesterified (Fig. 3i, *arrowhead*) HG were observed (Fig. 4a, d). The level of Ca^2+^-associated HG was significantly lower (Fig. 3j, *arrowhead*). Epitopes recognized by JIM7 and JIM5 had a specific distribution. Two layers of the zygote’s wall were visible. The inner layer of fertilized egg cell wall was continuous, while the outer, thicker layer was porous (Fig. 3h). In fertilized central cells, small clusters of fluorescence recognized by JIM7 were observed in the cytoplasm surrounding endosperm nuclei (Fig. 3g, *asterisks*).

The controls in which the primary antibodies were omitted showed a lack of fluorescence in the ovule. Only orange autofluorescence of the chalazal region of the embryo sac wall was observed (data not shown).

## Discussion

To date, little is known about the participation of HGs at the final stage of progamic phase, when the pollen tube changes its growth direction in the ovary to enter the micropyle. Our results revealed that the extracellular matrix of the ovule’s micropylar canal is subject to pollination-induced rearrangements. The micropylar canal derived from a mature, non-pollinated flower contained not only highly methyl esterified, but also Ca^2+^-associated HG, which is likely a component of the exudate. Pollination induced a decrease in latter fraction of pectins and an increase in the level of de-esterified HG. This result indicates the degradation of Ca^2+^-associated HG after pollination. This process takes place as early as 8 h after pollination, before the pollen tubes outgrow the style of the pistil. Hence, the signal inducing the degradation of this category of pectins precedes the physical contact between the male gametophyte and ovule’s micropylar canal. The degradation of ECM-stiffening, Ca^2+^-associated polygalacturonans presumably leads to the relaxation of the micropylar canal structure, which facilitates its penetration by pollen tubes. Previous studies have demonstrated the synthesis and exocytosis of the male gametophyte-specific enzymes that modify wall polysaccharides, including pectin methyl esterases (PMEs), polygalacturonases (PGs) and pectin lyases (Li et al. 2002; Bosh and Hepler 2005; Jiang et al. 2005; Tian et al. 2006; Röckel et al. 2008; Hepler et al. 2013).

The lysis of Ca^2+^-crosslinked HG has been described to be accompanied by the release of free calcium ions, which plays an important role in pollen tube’s elongation (Lenartowska et al. 2011). In all plants investigated to date: pearl millet (Chaubal and Reger 1992a), sunflower (Zhang et al. 1995), tobacco (Tian and Russel 1997), cotton (Zhang et al. 1997), *B. napus* (Yu et al. 1998), *Plumbago zeylenica* (Tian et al. 2000), and *Crocus* (Chichiriccó et al. 2002), the micropyle appears to contain abundant levels of calcium, which correlates closely with fertility (Chudzik and Snieżko 2003; for review, see Ge et al. 2007). The mechanism by which calcium environment at the micropyle is created is not fully understood. Our results suggest that in *H. orientalis*, Ca^2+^-associated pectins presumably comprise the extracellular storage sites of free calcium ions, which could be released to the ECM via pollination-induced degradation of HGs.

After fertilization, in the ovule’s micropylar canal, there was a second increase in Ca^2+^-bound HG level. This phenomenon likely facilitates the polyspermy block, which is blocking of other pollen tubes growth into the micropyle of already fertilized ovule. The binding of free calcium ions by HG is likely to lead to an immediate reduction of the Ca^2+^ concentration at the micropyle, which makes it more difficult for the subsequent pollen tubes to find the already fertilized ovule. Notwithstanding, further investigation involving the analysis of free calcium levels in hyacinth ovules would be needed for confirmation of this interpretation. In addition, the effect of HG chains cross-binding calcium ions is a stiff pectin gel, which mechanically impedes the penetration of the micropylar canal by pollen tubes.

Our experiments showed that the pattern of pectic composition of female germ unit’s apoplast (egg apparatus and the central cell) reflects the preparation of these cells for the entry of the pollen tube and fertilization.

After the elongation through the micropyle, the pollen tube penetrates one of the synergids, arrests its growth, and bursts to release two sperm cells (Huang and Russel 1992; Higashiyama 2002; Higashiyama et al. 2003; Li et al. 2009; Dresselhaus and Sprunck 2012). It has been well established that synergid cells and their filiform apparatus play essential role in pollen tube guidance and reception (Punwani et al. 2007; Punwani and Drews 2008; Márton and Dresselhaus 2010). We propose that pectins present in the filiform apparatus are likely involved in regulation of the interaction between synergid and male gametophyte. In the examined hyacinth before pollination, in the filiform apparatus there was a large pool of highly methyl-esterified HG and a lower amount of non-esterified HG, while Ca^2+^-associated HG was not found. Additionally, during the progamic phase, the filiform apparatus was almost completely devoid of Ca^2+^-associated HG; it contained only methyl-esterified and de-methyl-esterified HG. This result indicates that in the mature embryo sac, HG de-esterification was already initiated in the filiform apparatus; these HGs, however, do not bind Ca^2+^. This pattern of pectic composition of the filiform apparatus is in line with previous studies that showed that this structure is the site of a large pool of free and loosely bound calcium, which might play a role in facilitating the optimal growth environment for elongating pollen tubes (Chaubal and Reger 1992b, 1994; Huang and Russel 1992; Tian and Russel 1997; Yu et al. 1998; Tian et al. 2000; Dumas and Gaude 2006; Dresselhaus and Márton 2009; Dresselhaus and Franklin-Tong 2013). Additionally, a significant decrease in methyl-esterified HG during the progamic phase indicates that HG lysis takes place in the filiform apparatus, and the HG degradation products might be used by growing pollen tubes. It has been proposed that the filiform apparatus facilitates transport of substances into and out of the synergid; for example, it imports nutrients and exports pollen tube attractants that guide the pollen tube to the female gametophyte (Huang and Russel 1992; Higashiyama 2002; Punwani and Drews 2008; Márton and Dresselhaus 2010). Additionally, the degradation of unesterified HG induces the formation of oligogalacturonides that serve as signaling molecules (OGs) (Wolf et al. 2009).

In this study, fertilization of *H. orientalis* was followed by a change in pectic composition of the filiform apparatus. In this structure, apart from methyl-esterified and de-methyl-esterified HG, there was also a large pool of Ca^2+^-associated HG. We suggest that the latter HG fraction creates the first polyspermy barrier. As in the micropylar canal, the Ca^2+^-associated HG could stiffen the filiform apparatus and prevent penetration by additional pollen tubes.

After entering the synergid, the pollen tube releases its contents, and the sperm cells are transported to the area between the egg cell and the central cell. This region of the embryo sac becomes the site of fusion of the male gametes with target cells. In our research, these processes are preceded by a change in the pectin composition of the egg apparatus apoplast and the “micropylar” region of the central cell. In the mature embryo sac, the walls of synergids and egg cell contained mainly de-methyl-esterified HG, while Ca^2+^-associated HGs were not present. Thus, the de-methyl esterification of pectins in these cells walls was already initiated during the maturation of the embryo sac, although HGs did not form the “egg-box” structures. During the progamic phase, the apoplast of the egg cell and the adjoining wall of the central cell were devoid of this category of pectins. The lack of continuity of the cell wall in the region between the egg cell and the central cell has been observed in other plant species, including *Arabidopsis* (Mansfield et al. 1991; Kasahara et al. 2005) and *Zea mays* (Diboll and Larson 1966). It has been suggested that this discontinuity enables the communication and/or transport of nutrients between the cells (for review, see Punwani and Drews 2008). We showed that in *H. orientalis*, pollination induced a complete lysis of HG (and likely cell walls) in the region of the imminent transport to the male gametes from synergid cell and in the region of their fusion with target cells. Lysis of HG in this area of the embryo sac was strictly controlled as indicated by the observation that the apoplast of the antipodal cells in the same embryo sac still contained de-methyl-esterified HG, a substrate for polygalacturonases.

After fertilization, a new cell wall was synthesized around the zygote; this wall contained both highly methyl-esterified and de-methyl-esterified HG. Fertilization initiated HG synthesis, which is indicated by the observation, that only in the cytoplasm of the zygote did we find highly esterified HG, while it was not localized in the cytoplasm of unfertilized egg cell. In addition, the occurrence of Ca^2+^-associated HG in zygote wall indicated that HG synthesis, de-esterification and wall stiffening via crosslinking with Ca^2+^ was triggered rapidly and soon after fertilization. Similarly, in an in vitro study on *Z. mays* using electrically mediated fusion, Kranz et al. (1995) observed a release of the cell wall components from the egg cell 30 s after fusion with the sperm cell (Dumas and Gaude 2006; Spielman and Scott 2008). The rapid synthesis of this unique composition of the zygote cell wall is likely one of the mechanisms of isolation of the developing embryo from surrounding tissue. Additionally, this could comprise one of the polyspermy block events, which prevents penetration of more than one sperm cell into the female gamete (Spielman and Scott 2008). In this study on *H. orientalis,* newly synthesized wall around the zygote had a very distinctive pattern of HG localization, which we called “sporoderm-like” (Fig. 3h). Similar to the pollen grain sporoderm (Gołaszewska and Bednarska 1999), HGs were located in a continuous inner layer and in the area of the discontinuous “trabecule-like” outer layer. This specific structure of the wall may play an important role in the interaction between the zygote and other ovule cells such as controlling the flow of nutrients, signaling molecules (OGS) and metabolites between the zygote and the endosperm. In the central cell, fertilization also induces HG synthesis. In the cytoplasm surrounding the emerging endosperm cells, highly methyl-esterified HG was found (Fig. 3g, *asterisks*). This observation shows that after karyogamy, developing endosperm cells prepare for cellularization, by synthesis of highly methyl-esterified HG. This HG fraction is stored in the cytoplasm and may be used as a material for the construction of the endosperm cell walls.

In conclusion, our findings reveal that HGs might play an important role in sexual reproduction of *H. orientalis*. The rearrangements in the composition of examined HGs in the ovule’s ECM and the apoplast of female gametophyte cells, induced by pollination and fertilization, are crucial for the following events: (1) creation of an optimal environment for directed growth of pollen tubes and for final entry of the male gametophyte into the embryo sac, (2) preparation of the egg cell and the central cell for fusion with sperm cells, and (3) zygote isolation and polyspermy block.

## Abbreviations

ECM: Extracellular matrix
HGs: Homogalacturonans
JIM7 MAb: Monoclonal antibody against high methyl-esterified homogalacturonic acid
JIM5 MAb: Monoclonal antibody against weakly methyl-esterified homogalacturonic acid
2F4 MAb: Monoclonal antibody against calcium cross-linked homogalacturonic acid
